# Phosphatidic acid enhances mTOR signaling and resistance exercise induced hypertrophy

**DOI:** 10.1186/1743-7075-11-29

**Published:** 2014-06-16

**Authors:** Jordan M Joy, David M Gundermann, Ryan P Lowery, Ralf Jäger, Sean A McCleary, Martin Purpura, Michael D Roberts, Stephanie MC Wilson, Troy A Hornberger, Jacob M Wilson

**Affiliations:** 1 Department of Health Sciences and Human Performance, The University of Tampa, 401 W. Kennedy Blvd., Box 30 F, Tampa, FL 33606, USA; 2 Department of Comparative Biosciences, University of Wisconsin-Madison, Madison, WI, USA; 3 Increnovo LLC, 2138 E Lafayette Pl, Milwaukee, WI 53202, USA; 4 School of Kinesiology, Auburn University, Auburn, AL, USA; 5 Department of Nutrition, IMG Academy, Bradenton, FL, USA

**Keywords:** Supplementation, Skeletal muscle, Protein synthesis, Phospholipid, Ergogenic aid

## Abstract

**Introduction:**

The lipid messenger phosphatidic acid (PA) plays a critical role in the stimulation of mTOR signaling. However, the mechanism by which PA stimulates mTOR is currently unknown. Therefore, the purpose of this study was to compare the effects of various PA precursors and phospholipids on their ability to stimulate mTOR signaling and its ability to augment resistance training-induced changes in body composition and performance.

**Methods:**

In phase one, C_2_C_12_ myoblasts cells were stimulated with different phospholipids and phospholipid precursors derived from soy and egg sources. The ratio of phosphorylated p70 (P-p70-389) to total p70 was then used as readout for mTOR signaling. In phase two, resistance trained subjects (n = 28, 21 ± 3 years, 77 ± 4 kg, 176 ± 9 cm) consumed either 750 mg PA daily or placebo and each took part in an 8 week periodized resistance training program.

**Results:**

In phase one, soy-phosphatidylserine, soy-Lyso-PA, egg-PA, and soy-PA stimulated mTOR signaling, and the effects of soy-PA (+636%) were significantly greater than egg-PA (+221%). In phase two, PA significantly increased lean body mass (+2.4 kg), cross sectional area (+1.0 cm), and leg press strength (+51.9 kg) over placebo.

**Conclusion:**

PA significantly activates mTOR and significantly improved responses in skeletal muscle hypertrophy, lean body mass, and maximal strength to resistance exercise.

## Introduction

Increasing or maintaining skeletal muscle mass is an important target for a variety of populations ranging from athletes to the elderly. Skeletal muscle mass is largely dependent upon muscle protein synthesis (MPS), and a protein kinase called the mechanistic target of rapamycin (mTOR) has been widely recognized as a key regulator of muscle growth. Specifically, elevations in energy status [1-3], amino acids [4,5], and growth factors [6,7] can increase MPS through an mTOR-dependent mechanism. Furthermore, several studies have also shown that signaling by mTOR is required for mechanically-induced increases in MPS and the ultimate hypertrophic response [8-11].

Phosphatidic acid (PA) is a diacyl-glycerophospholipid, in which two fatty acids and a phosphate group are covalently bonded to a glycerol molecule through ester linkages. PA can act as a signaling lipid, it is a precursor for the biosynthesis of other lipids, and it is a major constituent of cell membranes. Recent studies have shown that mechanical stimuli can induce an increase in the intracellular levels of PA and that the increase in intracellular PA contributes to the activation of mTOR-dependent signaling events such as ribosomal S6 kinase 1 (p70) threonine 389 phosphorylation (P-p70-389) [12]. It has also been shown that PA can directly bind to the FKBP12 · rapamycin binding (FRB) domain of mTOR, and in doing so, it activates mTOR signaling [13,14]. It has also been shown that exogenous sources of PA can promote the activation of mTOR signaling, yet the effects of exogenous PA appear to be driven through multiple mechanisms. For example, Winter and colleagues [15] have demonstrated that the exogenous addition of PA to fibroblasts results in the activation of mTOR signaling via an indirect mechanism that is dependent on PA being metabolized to lysophosphatidic acid (LPA) and activating LPA family receptors. Furthermore, this study demonstrated that the activation of LPA receptors induces mTOR signaling via an ERK-dependent mechanism. Alternatively, You et al. [14] have shown that passively stretching skeletal muscles leads to an increase in intracellular PA and mTOR signaling and that the activation of mTOR signaling occurs through an ERK-independent mechanism. Collectively, these findings suggest that the exogenous provision of PA and mechanical stimuli can activate mTOR signaling through distinct pathways, and it is possible that the activation of these distinct pathways could have additive effects on mTOR signaling.

While PA plays a critical role in the stimulation of mTOR signaling and an increase in PA is sufficient for the activation of mTOR signaling, the exact mechanism by which PA stimulates mTOR is currently unconfirmed; although, it is very likely that PA primarily mediates its effects via direct binding to mTOR [13,14]. PA can be synthesized from a variety of reactions via multiple reactants, but it is not clear if other precursors (glycerol-3-phosphate (G3P), LPA or diacylglycerol (DAG)), or the addition of head groups to the PA molecule (phosphatidylcholine (PC), phosphatidylserine (PS), phosphatidylethanolamine (PE) or phosphatidylinositol (PI)), have a similar ability to activate mTOR signaling. Moreover, different sources of PA (soy, egg) can have varying degrees of unsaturated or saturated fatty acid chains and this can influence the behavior of PA. Specifically, it has been suggested that two saturated fatty acids will promote storage, but one saturated and one unsaturated fatty acid will promote signaling [16].

We have previously examined the absorption kinetics of 1.5 g PA and observed an increase in plasma concentration after 30 minutes. PA concentrations appear to plateau between 1 and 3 hours following ingestion while peaking at 3 hours following ingestion. After 7 hours, PA concentrations remained elevated. In addition, LPA demonstrated a bimodal absorption kinetic with plasma concentration peaking at 1 hour, returning to baseline at 2 hours, and peaking again at 3 hours [17]. Thus, exogenous elevations of PA may be provided through oral supplementation, while endogenous production could be fostered through a resistance training stimulus. Theoretically, the combination of the two could result in greater skeletal muscle hypertrophy than resistance training alone. However, to date, only one study has investigated the combination of oral PA supplementation combined with resistance training (RT). Specifically, Hoffman et al. [18] concluded that it is very likely that PA supplementation in humans undergoing progressive RT results in greater increases in squat strength and lean mass over the placebo. However, it is likely that this study was underpowered. Moreover, subjects in this pilot study were not supervised during RT. Finally, while the authors looked at indices of hypertrophy such as lean body mass, no direct measures of skeletal muscle hypertrophy were taken. Therefore, the results of whether or not PA supplementation enhances skeletal muscle hypertrophy were inconclusive. Thus, the purpose of this study was to compare the effects of various PA precursors on their ability to stimulate mTOR signaling and determine if any other phospholipid species are also capable of stimulating mTOR signaling. Following the initial investigation, we performed a double-blind, placebo-controlled study that was designed to assess the effects of orally administered PA on skeletal muscle hypertrophy, strength, and power when consumed during a periodized RT program. We hypothesized that PA supplementation would lead to increased improvements in strength, skeletal muscle hypertrophy, and power.

## Methods

### Phase 1 (cell culture tests)

C_2_C_12_ myoblasts (ATCC; Manassas, VA, USA) were plated at approximately 30% confluence and grown for 24 hours in 10% FBS High Glucose DMEM with antibiotics (100 μg/ml streptomycin and 100 U/ml penicillin; Sigma-Aldrich; St. Louis, MO, USA). At 16 hours prior to the experiment, myoblasts cells were switched to serum free high glucose DMEM (no antibiotics) and were approximately 70% confluent at the time of the experiment. All stimulants were dissolved in chloroform to yield a concentration of 10 mg/mL, with the exception of DAG which was dissolved at 2 mg/mL and G3P which was dissolved at 6 mg/mL. Each stimulant was then dried with a stream of nitrogen gas and resuspended in PBS to obtain either 20 or 60 nmol/100 μL, such that 100 μL added to 2 mL of media resulted in 10 or 30 μM respectively. Accordingly, cells were stimulated for 20 minutes with vehicle (Control; 100 μL of PBS) 10 or 30 μM of soy-derived (S) phosphatidylserine (S-PS, SerinAid®, Chemi Nutra, White Bear Lake, MN, USA), phosphatidylinositol (S-PI), phosphatidyl-ethanolamine (S-PE), phosphatidylcholine (S-PC), PA (S-PA, Mediator®, Chemi Nutra, White Bear Lake, MN, USA), lysophosphatidic acid (S-LPA), diacylglycerol (DAG), glycerol-3-phosphate (G3P), or egg-derived PA (E-PA). Cells were then harvested in lysis buffer (40 mM Tris, pH 7.5; 1 mM EDTA; 5 mM EGTA; 0.5% Triton X-100; 25 mM β-glycerophosphate; 25 mM NaF; 1 mM Na_3_VO_4_; 10 μg/mL leupeptin; and 1 mM PMSF) and subjected to immunoblotting with anti-phospho-p70 S6 Kinase (Thr389; Cell signaling #9234; 1:1000; Danvers, MA, USA) as previously described [19]. Once the appropriate image was captured, membranes were stripped for 30 minutes in stripping buffer (100 mM β-Mercaptoethanol, 2% SDS, 62.5 mM Tris HCL pH 6.8) maintained at 50°C. Membranes were washed with TBST, blocked with 5% powdered milk in TBST for 1 h, and then immunoblotted with anti-p70 S6 Kinase (cell signaling #2708, 1:2000; Danvers, MA, USA). Once the appropriate image was captured, the membranes were stained with Coomassie Blue to verify equal loading in all lanes. Densitometric measurements were performed by determining the density of each band using the public domain ImageJ software (U.S. National Institutes of Health, Bethesda, MD, USA; http://rsb.info.nih.gov/nih-image/). The ratio of P-p70-389 to total p70 was used as readout for mTOR signaling. C_2_C_12_ myoblasts were chosen, as it has previously been established that changes in P-p70-389 phosphorylation are a valid marker of PA induced changes in mTOR signaling [20].

### Phase 2 (human efficacy study)

Thirty-four males were recruited from the University of Tampa to participate in this study. Twenty eight males (21 ± 3 years, 77 ± 7 kg, 176 ± 9 cm) were used for data analysis. Three subjects elected to discontinue their participation prior to beginning the intervention, citing a more demanding schedule than they had anticipated; two subjects ceased participation, claiming the resistance training protocol was too onerous for them to complete; and one subject was removed from data analysis due to a failure to comply with the prescribed diet. Subjects were equally divided into the PA (n = 14, 78 ± 9 kg, 177 ± 7 cm) and PLA (n = 14, 76 ± 6 kg, 175 ± 11 cm) groups. All participants were required to abstain from consuming any muscle-building supplements (e.g. creatine) for 1 month prior to pretest measures, be non-smokers, have RT experience of no less than one year, and have participated in RT at least three days per week for the past six months to be included in this study. Participants were allowed, although not provided with, multivitamin and protein powder supplementation during the month prior to the initiation of the study. Participants were carefully matched according to their lean body mass (LBM), rectus femoris cross sectional area (CSA), and leg press 1-repetition-maximum (1RM), and they were then equally divided into either the PA or placebo (PLA) groups. Measures of leg press and bench press 1RM, LBM, fat mass, total mass, and CSA were taken prior to, and following, the RT protocol.

RT occurred three days per week with 48–72 hours between RT sessions. Each body part was trained 1–2 times per week following a daily undulating periodized scheme. Each participant performed a 5 RM for each exercise prior to the first four weeks with the exception of the bench press and leg press, in which true 1RM values were determined. 5 RM testing was repeated at the end of week 4 for the new exercises. The 1RM testing protocol consisted of 1 set of 10–12 repetitions at approximately 50% 1RM followed by 1 set of 2–3 repetitions at approximate intensities of 75% and 85% 1RM. After the final warm up set, weight was increased in 5-20 lb increments until 1RM was attained. 5RM determination followed an identical pattern; however, intensities were relative to 5RM instead of 1RM. These RM values were used to calculate the load used for each exercise for each participant. These exercises were also altered at week 5 to introduce a more novel stimulus. All participants were required to perform the prescribed number of repetitions with their prescribed weight. In the event that a subject reached muscular failure, a laboratory researcher assisted with the completion of the exercise. A comprehensive outline of the workouts can be found in Table 1.

**Table 1:** Workout program

**Monday**		**Wednesday**		**Friday**			**Monday & Wednesday repetitions**	**Friday repetitions**	**Monday & Wednesday rest**	**Friday rest**
**Week 1-4**	**Week 4-8**	**Week 1-4**	**Week 4-8**	**Week 1-4**	**Week 4-8**					
Leg press	Leg press	Bent over row	Pendlay row	Leg press	Leg press	**Week 1**	12	5	45 s	3-5 m
Leg extension	safety bar squat	Barbell shrug	Hexbar shrug	Bench press	Bench press	**Week 2**	10	3	60s	3-5 m
Leg curl	barbell lunge	Straight arm pulldown	Pulldown	Leg extension	Safety bar squat	**Week 3**	8	2	90s	3-5 m
Hyperextion	Stiff leg deadlift	Australian row	Decline dumbell row	Close grip bench press	Flat dumbell press	**Week 4**	6	1	120 s	3-5 m
Bench press	Bench press	Barbell shoulder press	Dumbell shoulder press			**Week 5**	12	5	60s	3-5 m
Incline dumbell press	Flat dumbell press	Isolated barbell military	Upright row			**Week 6**	10	3	60s	3-5 m
Close grip bench press	Cable crossover	Dumbell lateral raise	Barbell front raise			**Week 7**	8	2	90s	3-5 m
Cable rope extensions	Skull crushes	Dumbell bicep curls	Barbell bicep curls			**Week 8**	6	1	120 s	3-5 m

The repetition scheme provided was used for all of the exercises on the individual day indicated.

Strength was assessed via 1-RM testing of the leg press and bench press. Total strength was calculated as the sum of both leg press and bench press. Body composition (lean body mass, fat mass, and total mass) was determined on a Lunar Prodigy DXA apparatus (software version, enCORE 2008, Madison, Wisconsin, U.S.A.). Skeletal muscle hypertrophy was determined via changes in CSA of the rectus femoris at 50% femur length while the participant rested supine with a portable ultrasound device (Logiq e, General Electric Medical Systems, Milwaukee, WI, USA) in B mode with a wide-band linear array transducer (12 L-RS, General Electric Medical Systems, Milwaukee, WI, USA) sampling at 12 MHz. Ultrasound has previously been verified as a valid measurement of CSA [21]. However in the present study, a simpler, conventional method was used as opposed to the panoramic method used by Ahtiainen et al. Using the conventional method, a single image is captured for subsequent measurement instead of combing several images along a path, as with panoramic. Femur length was defined as the distance between the anterior superior iliac spine and the superior aspect of the patella. Probe placement was traced with permanent marker, and each subject was instructed to reapply the mark daily. Additionally, researchers affirmed and reapplied the mark during each laboratory and resistance training visit. Upon retesting, 50% femur length was remeasured to assure the mark had not deviated. The average of the greatest two out of three measurements was used to CSA value. Power was assessed during a maximal cycling ergometry test. During the cycling test, the volunteer was instructed to cycle against a predetermined resistance (7.5% of body weight) as fast as possible for 10 seconds [22]. The saddle height was adjusted to the individual’s height to produce a 5–10° knee flexion while the foot was in the low position of the central void. A standardized verbal stimulus was provided to the subjects. Power output was recorded in real time by a computer connected to the Monark standard cycle ergometer (Monark model 894e, Vansbro, Sweden) during the 10-second sprint test. Peak power (PP) was recorded using Monark Anaerobic test software (Monark Anaerobic Wingate Software, Version 1.0, Monark, Vansbro, Sweden). From completion of Wingate tests performed over several days, interclass correlation coefficient for peak power was 0.96.

Two weeks prior to and throughout the study, subjects were placed on a diet consisting of 25% protein, 50% carbohydrates, and 25% fat by a registered dietician who specialized in sport nutrition. Subjects met as a group with the dietitian, and they were given individual meal plans at the beginning of the study. Daily total of calories were determined by the Harris Benedict equation and tracked by weekly logs to ensure compliance. The PA group received 750 mg of soy-derived PA (Mediator®, Chemi Nutra, White Bear Lake, MN) per day, while the PLA group received 750 mg of rice flour, each delivered in 5 visually identical capsules. On RT days, participants consumed 450 mg of their respective supplement 30 minutes prior to RT and 300 mg immediately following RT with 24 g of hydrolyzed collagen protein powder from beef skin (Peptiplus XB agglomerated, Gelita AG, Eberbach, Germany) mixed with 500 ml water. The protein supplement was provided in order to ensure control for post-exercise meals between groups and hydrolyzed collagen was chosen as an incomplete protein source low in leucine (3.2 weight%). On non-RT days, participants consumed 450 mg of their respective supplement with breakfast and the remaining 300 mg with dinner. Participants were required to return to the laboratory with their empty bags to ensure compliance. Post-study analysis of the subjects’ diet revealed that it consisted of 25% protein, 51% carbohydrates, and 24% fat, with no differences between groups. The PA group consumed 2,865 ± 271 Calories per day (79.5 ± 6.7 g fat; 358.1 ± 28.6 g carbohydrate; 179.0 ± 15.0 g protein), and the PLA group consumed 2,795 ± 236 Calories per day (74.5 ± 5.4 g fat; 356.4 ± 32.1 g carbohydrate; 174.6 ± 12.9 g protein). Since the dietician had weekly interaction with the subjects, all subjects remained compliant throughout the study. Product formulations were blinded to both the investigators and the volunteers and coded so that neither knew which formulation was consumed during each trial.

All participants were informed of all risks associated with the study, and each participant signed an informed consent prior to beginning the study. This investigation was approved by the University’s Institutional Review Board.

#### Statistics

Phase 1 data was analyzed using a one way analysis of variance (ANOVA) for all data. A Tukey’s Multiple Comparison Test was used to determine significant differences between treatments. Significance was set at *P* < 0.05. Statistical analyses were performed on SigmaStat software (San Jose, CA, USA). A repeated measures ANOVA model was used to measure group, time, and group by time interactions in phase 2. If any main effects were observed, a Tukey post-hoc was employed to locate where differences occurred. Data are presented as mean ± standard deviation. All phase 2 statistics were run using Statistica software (Statsoft, Tulsa, OK, USA).

## Results

### Cell culture tests

**Figure 1:**
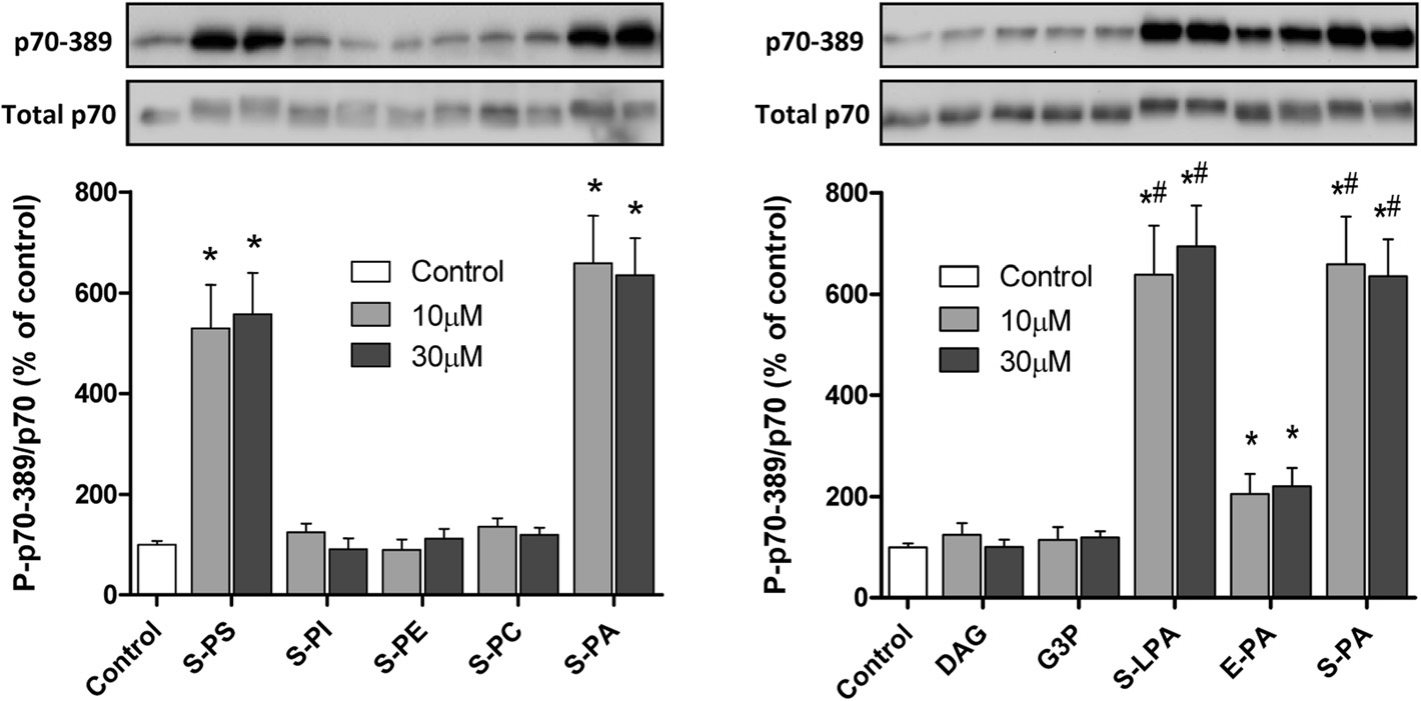
**The effect of various lipids on the activation of mTOR signaling.** C_2_C_12_ myoblasts were stimulated for 20 minutes with vehicle (Control), or 10-30 μM of soy-derived (S) phosphatidylserine (S-PS), phosphatidylinositol (S-PI), phosphatidyl-ethanolamine (S-PE), phosphatidylcholine (S-PC), PA (S-PA), lysophosphatidic acid (S-LPA), diacylglycerol (DAG), glycerol-3-phosphate (G3P), or egg-derived PA (E-PA). The samples were then subjected to Western blot analysis for p70 phosphorylated on the threonine 389 residue (p70-389) and total p70. The ratio of these signals was calculated and used as a marker of mTOR signaling. Values in the graphs represent the mean + SEM and were obtained from 2–3 independent experiments (n = 4–12/group). * Significantly different from control (*p* < 0.001). ^#^ Significantly different from E-PA within each respective dose (*p* < 0.001).

As shown in Figure 1, S-PI, S-PE, S-PC, DAG, and G3P elicited no increase in the ratio of P-p70-389 to total p70 compared to vehicle stimulated cells. In contrast, elevated mTOR signaling was observed at all tested concentrations of S-PS (529, and 558%), E-PA (206, and 221%), S-LPA (638, and 694%), and S-PA (658, and 636%; p < 0.05). In addition, S-LPA and S-PA increased mTOR signaling to a greater degree than did E-PA at all concentrations (p < 0.05).

### Body composition

**Figure 2:**
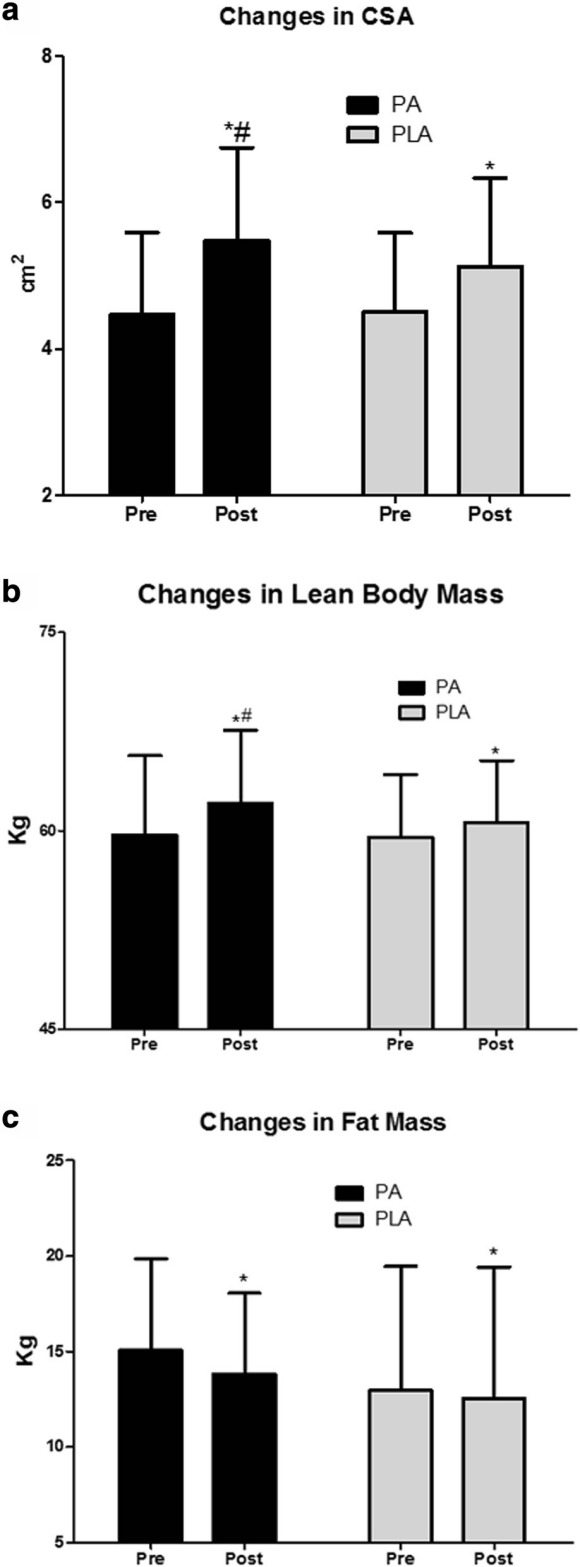
**Changes in body composition. a**. PA significantly improves the resistance training-induced increase in CSA following 8 weeks of periodized resistance training. Data presented are means and standard deviations. (*denotes significantly different from pre, ^#^ denotes significantly different from placebo). **b**. PA significantly improves the resistance training-induced increase in LBM following 8 weeks of periodized resistance training. Data presented are means and standard deviations. (*denotes significantly different from pre, ^#^ denotes significantly different from placebo). **c**. PA and PLA both experience significant fat loss following 8 weeks of periodized resistance training. However, only a trend is observed between groups over time. (*denotes significantly different from pre).

No differences existed between groups at baseline for any measure. There was a significant group x time effect (p = 0.02) for CSA (Figure 2a), in which the PA group increased (pre 4.5 ± 1.1 cm^2^, post 5.5 ± 1.3 cm^2^, Effect Size (ES) = 0.92) to a greater extent than the PLA group (pre 4.5 ± 1.1 cm^2^, post 5.1 ± 1.2 cm^2^, ES = 0.52). There was a significant group × time effect (p = 0.01) for LBM (Figure 2b), in which the PA group increased to a greater extent (pre 59.7 ± 6.0 kg, post 62.1 ± 5.5 kg, ES = 0.42) than the PLA group (pre 59.5 ± 4.7 kg, post 60.7 ± 4.7 kg, ES = 0.26). There was a significant time effect (p = 0.02) for Total Body Mass (TBM) in which the PA group increased from 78.1 ± 8.7 to 78.7 ± 7.9 kg and the PLA group increased from 75.7 ± 5.8 to 76.5 ± 6.1 kg, but no differences existed between groups (p = 0.71). There was a significant time effect (p < 0.01) for fat mass (Figure 2c), in which there was a trend (p = 0.068) for fat mass to decrease to a greater extent in the PA group (pre 15.1 ± 4.8 kg, post 13.8 ± 4.2 kg, ES = −0.28) than the PLA group (pre 13.0 ± 6.5 kg, post 12.5 ± 6.9 kg, ES = −0.07).

### Strength and power

**Figure 3:**
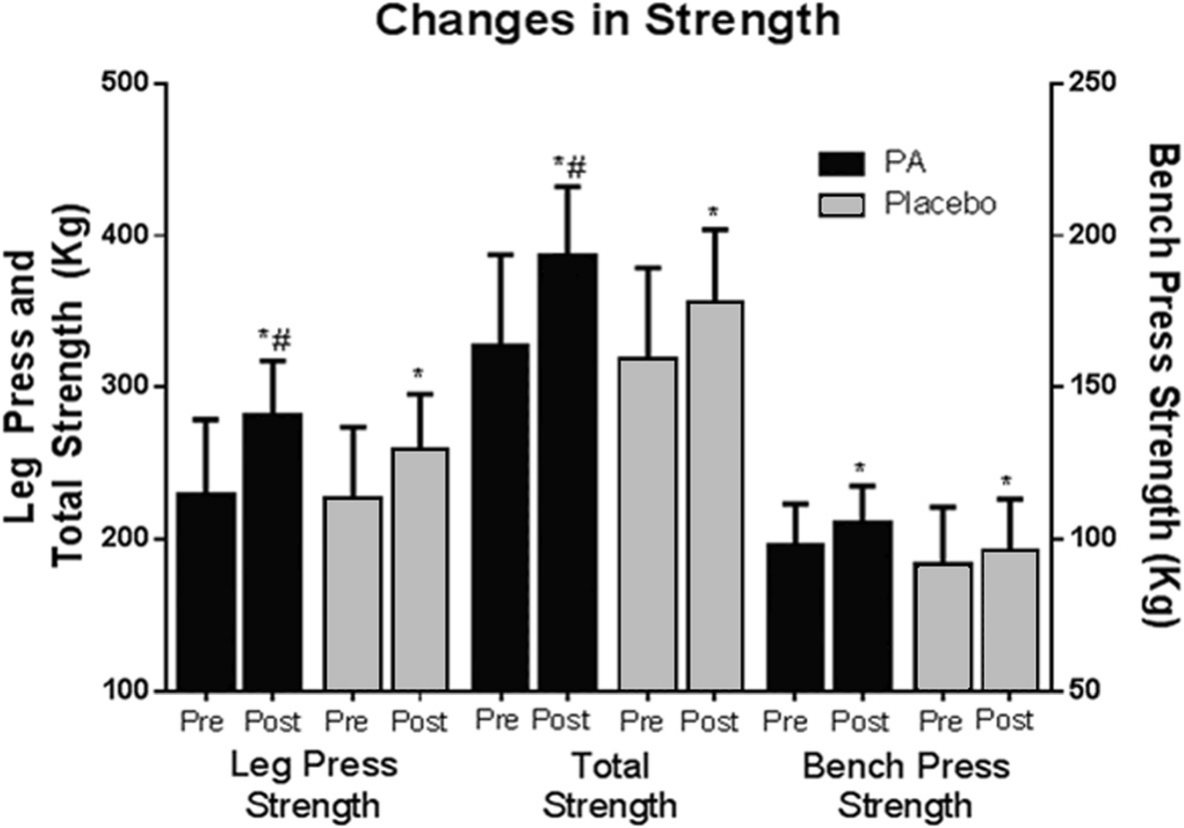
**Changes in muscular strength.** Changes in muscle strength from pre to post intervention are indicated by 1-repetition maximum (1RM) measurements of leg press and bench press. Total strength is calculated by the summation of the two measurements. (*denotes significantly different from pre, ^#^ denotes significantly different from placebo).

There was a significant group x time effect (p < 0.05) for leg press 1RM, in which the PA group increased to a greater extent (pre 228.7 ± 49.5 kg, post 280.6 ± 36.2 kg, ES = 1.2) than the PLA group (pre 226.3 ± 47.2 kg, post 258.7 ± 36.1 kg, ES = 0.78). There was a significant time effect (p < 0.01) for bench press 1RM, in which both the PA (pre 98.0 ± 13.5 kg, post 105.0 ± 12.4 kg, ES = 0.5) and PLA (pre 91.4 ± 19.1 kg, post 96.1 ± 17.0 kg, ES = 0.25) increased; however, no differences were present between groups (p = 0.11). There was a significant group x time effect (p < 0.05) for total strength, in which the PA group increased to a greater extent (pre 327.4 ± 59.9 kg, post 386.4 ± 45.8 kg, ES = 1.1) than the PLA group (pre 318.3 ± 60.3, post 355.5 ± 48.4, ES = 0.68). Change values for strength can be found in Figure 3. There was a significant time effect (p < 0.01) for Wingate peak power, which increased in the PA group from 760.5 ± 166.0 W to 822.8 ± 217.3 W and from 733.5 ± 105.8 to 797.3 ± 122.3 W in the PLA group; however, there were no differences between groups (p = 0.97).

## Discussion

The purpose of this study was to screen for the most effective phospholipid based activator of mTOR signaling and to investigate the effects of PA on human skeletal muscle hypertrophy, LBM, strength, and power when consumed during a RT program in a double-blind, placebo-controlled design. Our hypotheses were that PA supplementation would augment the increase in skeletal muscle hypertrophy, strength, and power that is induced by RT.

mTOR is a master regulator of cellular growth, and PA is widely known to be involved in the regulation of the mTOR pathway [13,20,23,24]. Specifically, our previous studies have determined that an increase in the exogenous availability of C8 PA, or PA derived from egg, is sufficient to induce a significant increase in mTOR signaling [14]. Research by Lehman et al. reported the effects of PA along with various other phospholipids in their ability to induce p70 activity revealing that only PA was sufficient to induce an increase in p70 activity [25]. However, phase one of the current study demonstrates that not only is soy-derived PA sufficient, but soy-derived LPA and S-PS can also stimulate a robust increase in mTOR signaling. Moreover, these lipid species were directly compared to the effectiveness of E-PA, and the results demonstrated that, compared to egg-derived lipids, all of these soy-derived lipids were more potent agonists of mTOR signaling. This could be due to the composition of the PA molecule. For instance, Foster et al. have reported differing effects of PA based upon the structure of its fatty acid components; wherein, two saturated fatty acids promoted storage, yet one saturated and one unsaturated fatty acid promoted signaling [16]. Thus, the higher unsaturated fat content of soy may explain these observations. This suggests that soy derived PA, LPA, and PS are superior agonists of mTOR signaling, and therefore, they appear to be suitable candidates for augmenting the effects of RT on muscle mass. However, it should be noted that reacylation of PA after absorption is dependent upon the nutritional status of the individual, and therefore, the observed differences between the sources of PA (egg and soy) may or may not translate to the effects observed with oral administration of PA.

PA can be generated by G3P, one de novo fatty acid, and one fatty acid from the diet via a lysophosphatidic acid acyltransferase (LPAAT) mediated mechanism, or by phosphorylation of DAG via DAG kinase, or by hydrolysis of PC through phospholipase D (PLD). Although PC, DAG and G3P did not directly activate mTOR signaling in our cell culture studies, it remains possible that chronic oral administration could potentially increase PA pools by acting as a substrate for PA synthesis. Consistent with this possibility, previous studies have shown that certain phospholipids can improve athletic performance [26]. For example, PC increases endurance by preventing exercise induced declines in choline levels and PS by blunting exercise induced increases in cortisol [27] and improving mental performance under stress [28]. However, no study has yet investigated the effects of long-term PS supplementation in combination with resistance exercise on potential gains in muscular hypertrophy. It is also important to note that although we have determined maximal mTOR activation in response to PA occurs at approximately 20 minutes, this is only one time point, and activation prior to, or following, 20 minutes could have been missed. Therefore, future research should investigate additional time points, as well as lower concentrations, as 10 μM appeared to be a saturating dose.

### Effects of PA supplementation on CSA and LBM

For decades, it has been well documented that RT leads to increases in muscle mass [29]. However, the mechanism for muscle hypertrophy due to a mechanical stimulus has only just begun to be elucidated. Recent research indicates that the lipid second messenger, PA, could be at least partially responsible for translating the mechanical stimulus of RT into the chemical signal for skeletal muscle hypertrophy [23]. For example, research conducted by O’Neil et al. [20] has demonstrated that the PA content of the cell is increased following eccentric contractions and this effect is associated with a robust activation of mTOR signaling.

It is interesting to note that the endogenously produced PA can directly bind to, and activate, mTOR signaling which, in-turn, can promote an increase in protein synthesis. These effects appear to be independent of the PI3K and ERK signaling pathways [14,20]. However, exogenous PA can also be hydrolyzed to LPA, which binds to the LPA family of G-protein-coupled receptors. The binding of LPA to these receptors results in a cascade of events which stimulate the ERK signaling pathway and also increase PLD activity within the cell [15]. The result is a dual mediated increase in mTOR signaling via increased PA content within the cell as well as via the activation of the ERK signaling pathway [15]. Thus through the activation of multiple pathways, supplementing with PA could result in greater rates of MPS than RT alone and might explain why we observed a greater increase in skeletal muscle accretion when supplementing with PA.

While a pilot study [18] found that changes in LBM were likely enhanced by PA supplementation, they did not find as robust of an effect as the present study in either the control or experimental conditions. For example, their results found no significant change in LBM in the placebo group (+0.1 kg LBM), and they found smaller effects in the experimental group (+1.7 kg LBM) compared to the present study. These findings may indicate that the training stimulus was inadequate to enhance LBM by itself, and it may indicate that changes in LBM were primarily driven by the supplement itself. It is also possible that these findings are the result of unsupervised training as research demonstrates greater increases in neuromuscular adaptations in supervised, as compared to unsupervised, training [30]. In contrast, the present study found increases in both LBM and hypertrophy in both the placebo and PA conditions. These findings may indicate that skeletal muscle hypertrophy was driven by both endogenous (training) and exogenous (PA supplementation) mechanisms. Therefore, future research may be interested in examining the effects of PA on muscle hypertrophy in the absence of a RT stimulus. Additionally, subjects were administered a collagen protein supplement in order to standardize for post-exercise nutrition. It should be noted, however, that there is a low leucine content in collagen protein. Therefore, we posit that collagen supplementation likely had minimal effects in stimulating training-induced responses in strength and body composition, as leucine is primarily responsible for protein’s effects on muscle protein synthesis [31]. We chose a collagen protein supplement to minimize possible effects of protein supplementation. Moreover while mTOR and P-p70-389 influence MPS, MPS was not directly measured in this study, and this mechanism for muscle hypertrophy must be validated in future research.

It is interesting to note that there was a trend (p = 0.068) for PA to decrease body fat (ES = 0.28). Past research strongly suggests that RT alone does not provide a strong stimulus for fat loss [32]. However since muscle mass is a very metabolically active tissue [33], the fat loss may be explained by the increased LBM. While we presently can only speculate as to why or if this may be the case, future research may be interested in exploring the possibility of PA as a fat loss agent, as PA is known to interact with many complexes [34].

### Strength and power

Strength is one of the most critical attributes underlying success in sport [35,36]. The collective results of the present study, as well as those from Hoffman [18], suggest that changes in strength following supervised and non-supervised RT are enhanced by PA supplementation. The observed increases in skeletal muscle CSA certainly contribute to the increases observed in strength, and as PA is not expected to directly increase strength, we believe the increases in strength are due to the increases in CSA, which has been well documented [29]. Additionally, we believe no differences were observed for power as the participants did not train with a power-oriented stimulus. Rather they trained for hypertrophy twice per week, and strength once per week. Thus, further research will be needed to determine if PA can enhance improvements in power.

## Conclusions

PA induces an increase in mTOR signaling in a cell culture model. Oral PA administration is capable of enhancing the anabolic effects of resistance training and contributes to muscle accretion over time. Oral PA supplementation can directly augment changes in skeletal muscle hypertrophy following a chronic RT stimulus and results in significant increases in strength and LBM over placebo.

## Competing interests

RJ and MP are independent paid consultants to Chemi Nutra and have been named as inventors on pending patents by Chemi Nutra. MP and RJ were not involved in data collection or analysis. All other authors declare that they have no competing interests.

## Authors’ contributions

Cell culture tests were performed by DMG and TAH at the University of Wisconsin-Madison. All human work was conducted at The University of Tampa by JMJ, RPL, SAM, and JMW. Dietary protocols were developed at the IMG Performance Institute by SMCW. RJ, MDR, and MP were involved in concept and design of the overall study and manuscript preparation. The manuscript was written through contributions of all authors. All authors have given approval to the final version of the manuscript.

## References

[B1] Bolster DR Crozier SJ Kimball SR Jefferson LS AMP-activated protein kinase suppresses protein synthesis in rat skeletal muscle through down-regulated mammalian target of rapamycin (mTOR) signaling J Biol Chem 2002 277 23977 23980 10.1074/jbc.C200171200 11997383

[B2] Inoki K Zhu T Guan KL TSC2 mediates cellular energy response to control cell growth and survival Cell 2003 115 577 590 10.1016/S0092-8674(03)00929-2 14651849

[B3] Hardie DG Hawley SA Scott JW AMP-activated protein kinase–development of the energy sensor concept J Physiol 2006 574 7 15 10.1113/jphysiol.2006.108944 16644800 PMC1817788

[B4] Anthony JC Yoshizawa F Anthony TG Vary TC Jefferson LS Kimball SR Leucine stimulates translation initiation in skeletal muscle of postabsorptive rats via a rapamycin-sensitive pathway J Nutr 2000 130 2413 2419 11015466 10.1093/jn/130.10.2413

[B5] Dickinson JM Fry CS Drummond MJ Gundermann DM Walker DK Glynn EL Timmerman KL Dhanani S Volpi E Rasmussen BB Mammalian target of rapamycin complex 1 activation is required for the stimulation of human skeletal muscle protein synthesis by essential amino acids J Nutr 2011 141 856 862 10.3945/jn.111.139485 21430254 PMC3077888

[B6] Dardevet D Sornet C Vary T Grizard J Phosphatidylinositol 3-kinase and p70 s6 kinase participate in the regulation of protein turnover in skeletal muscle by insulin and insulin-like growth factor I Endocrinology 1996 137 4087 4094 8828461 10.1210/endo.137.10.8828461

[B7] Frost RA Lang CH Differential effects of insulin-like growth factor I (IGF-I) and IGF-binding protein-1 on protein metabolism in human skeletal muscle cells Endocrinology 1999 140 3962 3970 10465265 10.1210/endo.140.9.6998

[B8] Hornberger T Stuppard R Conley K Fedele M Fiorotto M Chin E Esser K Mechanical stimuli regulate rapamycin-sensitive signalling by a phosphoinositide 3-kinase-, protein kinase B-and growth factor-independent mechanism Biochem J 2004 380 795 804 10.1042/BJ20040274 15030312 PMC1224227

[B9] Kubica N Bolster DR Farrell PA Kimball SR Jefferson LS Resistance exercise increases muscle protein synthesis and translation of eukaryotic initiation factor 2Bϵ mRNA in a mammalian target of rapamycin-dependent manner J Biol Chem 2005 280 7570 7580 10.1074/jbc.M413732200 15591312

[B10] Drummond MJ Fry CS Glynn EL Dreyer HC Dhanani S Timmerman KL Volpi E Rasmussen BB Rapamycin administration in humans blocks the contraction‒induced increase in skeletal muscle protein synthesis J Physiol 2009 587 1535 1546 10.1113/jphysiol.2008.163816 19188252 PMC2678224

[B11] Bodine SC Stitt TN Gonzalez M Kline WO Stover GL Bauerlein R Zlotchenko E Scrimgeour A Lawrence JC Glass DJ Akt/mTOR pathway is a crucial regulator of skeletal muscle hypertrophy and can prevent muscle atrophy in vivo Nat Cell Bio 2001 3 1014 1019 10.1038/ncb1101-1014 11715023

[B12] You JS Lincoln HC Kim CR Frey JW Goodman CA Zhong XP Hornberger TA The role of diacylglycerol kinase zeta and phosphatidic acid in the mechanical activation of Mammalian Target of Rapamycin (mTOR) signaling and skeletal muscle hypertrophy J Biol Chem 2013 289 1551 1563 24302719 10.1074/jbc.M113.531392 PMC3894336

[B13] Fang Y Vilella-Bach M Bachmann R Flanigan A Chen J Phosphatidic acid-mediated mitogenic activation of mTOR signaling Science 2001 294 1942 1945 10.1126/science.1066015 11729323

[B14] You JS Frey JW Hornberger TA Mechanical stimulation induces mTOR signaling via an ERK-independent mechanism: implications for a direct activation of mTOR by phosphatidic acid PLoS One 2012 7 e47258 10.1371/journal.pone.0047258 23077579 PMC3471816

[B15] Winter JN Fox TE Kester M Jefferson LS Kimball SR Phosphatidic acid mediates activation of mTORC1 through the ERK signaling pathway Am J Physiol Cell Physiol 2010 299 C335 C344 10.1152/ajpcell.00039.2010 20427710 PMC2928642

[B16] Foster DA Regulation of mTOR by phosphatidic acid? Cancer Res 2007 67 1 4 10.1158/0008-5472.CAN-06-3016 17210675

[B17] Purpura M Jäger R Joy JM Lowery RP Moore JD Wilson JM Effect of oral administration of soy-derived phosphatidic acid on concentrations of phosphatidic acid and lyso-phosphatidic acid molecular species in human plasma J Int Soc Sports Nutr 2013 10 P22 10.1186/1550-2783-10-S1-P22

[B18] Hoffman JR Stout JR Williams DR Wells AJ Fragala MS Mangine GT Gonzalez AM Emerson NS McCormack WP Scanlon TC Purpura M Jäger R Efficacy of phosphatidic acid ingestion on lean body mass, muscle thickness and strength gains in resistance-trained men J Int Soc Sports Nutr 2012 9 47 10.1186/1550-2783-9-47 23035701 PMC3506449

[B19] Frey JW Farley EE O'Neil TK Burkholder TJ Hornberger TA Evidence that mechanosensors with distinct biomechanical properties allow for specificity in mechanotransduction Biophys J 2009 97 347 356 10.1016/j.bpj.2009.04.025 19580773 PMC2711371

[B20] O'Neil TK Duffy LR Frey JW Hornberger TA The role of phosphoinositide 3-kinase and phosphatidic acid in the regulation of mammalian target of rapamycin following eccentric contractions J Physiol 2009 587 3691 3701 10.1113/jphysiol.2009.173609 19470781 PMC2742291

[B21] Ahtiainen JP Hoffren M Hulmi JJ Pietikainen M Mero AA Avela J Hakkinen K Panoramic ultrasonography is a valid method to measure changes in skeletal muscle cross-sectional area Eur J Appl Physiol 2010 108 273 279 10.1007/s00421-009-1211-6 19777252

[B22] Smith JC Fry AC Weiss LW Li Y Kinzey SJ The effects of high-intensity exercise on a 10-second sprint cycle test J Strength Cond Res 2001 15 344 348 11710663

[B23] Hornberger TA Chu WK Mak YW Hsiung JW Huang SA Chien S The role of phospholipase D and phosphatidic acid in the mechanical activation of mTOR signaling in skeletal muscle Proc Natl Acad Sci 2006 103 4741 4746 10.1073/pnas.0600678103 16537399 PMC1450240

[B24] Yoon M-S Sun Y Arauz E Jiang Y Chen J Phosphatidic acid activates mammalian target of rapamycin complex 1 (mTORC1) kinase by displacing FK506 binding protein 38 (FKBP38) and exerting an allosteric effect J Biol Chem 2011 286 29568 29574 10.1074/jbc.M111.262816 21737445 PMC3190997

[B25] Lehman N Ledford B Di Fulvio M Frondorf K McPhail LC Gomez-Cambronero J Phospholipase D2-derived phosphatidic acid binds to and activates ribosomal p70 S6 kinase independently of mTOR FASEB J 2007 21 1075 1087 10.1096/fj.06-6652com 17242159

[B26] Jäger R Purpura M Kingsley M Phospholipids and sports performance J Int Soc Sports Nutr 2007 4 5 10.1186/1550-2783-4-5 17908342 PMC1997116

[B27] Starks MA Starks SL Kingsley M Purpura M Jäger R The effects of phosphatidylserine on endocrine response to moderate intensity exercise J Int Soc Sports Nutr 2008 5 11 10.1186/1550-2783-5-11 18662395 PMC2503954

[B28] Jäger R Purpura M Geiss KR Weiss M Baumeister J Amatulli F Schroder L Herwegen H The effect of phosphatidylserine on golf performance J Int Soc Sports Nutr 2007 4 23 10.1186/1550-2783-4-23 18053194 PMC2217563

[B29] Young A Stokes M Round JM Edwards RH The effect of high-resistance training on the strength and cross-sectional area of the human quadriceps Eur J Clin Invest 1983 13 411 417 10.1111/j.1365-2362.1983.tb00122.x 6416856

[B30] Mazzetti SA Kraemer WJ Volek JS Duncan ND Ratamess NA Gomez AL Newton RU Hakkinen K Fleck SJ The influence of direct supervision of resistance training on strength performance Med Sci Sports Exerc 2000 32 1175 1184 10.1097/00005768-200006000-00023 10862549

[B31] Norton LE Wilson GJ Layman DK Moulton CJ Garlick PJ Leucine content of dietary proteins is a determinant of postprandial skeletal muscle protein synthesis in adult rats Nutr Metab (Lond) 2012 9 67 10.1186/1743-7075-9-67 22818257 PMC3488566

[B32] Wilson JM Marin PJ Rhea MR Wilson SM Loenneke JP Anderson JC Concurrent training: a meta-analysis examining interference of aerobic and resistance exercises J Strength Cond Res 2012 26 2293 2307 10.1519/JSC.0b013e31823a3e2d 22002517

[B33] Kleiber M Body size and metabolic rate Physiol Rev 1947 27 511 541 20267758 10.1152/physrev.1947.27.4.511

[B34] Jang J-H Lee CS Hwang D Ryu SH Understanding of the roles of phospholipase D and phosphatidic acid through their binding partners Prog Lipid Res 2012 51 71 81 10.1016/j.plipres.2011.12.003 22212660

[B35] Robbins DW Docherty D Effect of loading on enhancement of power performance over three consecutive trials J Strength Cond Res 2005 19 898 902 16287358 10.1519/R-15634.1

[B36] Wilson JM Duncan NM Marin PJ Brown LE Loenneke JP Wilson SM Jo E Lowery RP Ugrinowitsch C Meta-analysis of post activation potentiation and power: effects of conditioning activity, volume, gender, rest periods, and training status J Strength Cond Res 2013 27 854 859 10.1519/JSC.0b013e31825c2bdb 22580978

